# PD-L1 expression and *CD274* gene alteration in triple-negative breast cancer: implication for prognostic biomarker

**DOI:** 10.1186/s40064-016-2513-x

**Published:** 2016-06-21

**Authors:** Lei Guo, Wenbin Li, Xinxin Zhu, Yun Ling, Tian Qiu, Lin Dong, Yi Fang, Hongying Yang, Jianming Ying

**Affiliations:** Department of Pathology, National Cancer Center/Cancer Hospital, Chinese Academy of Medical Sciences and Peking Union Medical College, Panjiayuan Nanli 17#, Beijing, 100021 China; Department of Breast Surgical Oncology, National Cancer Center/Cancer Hospital, Chinese Academy of Medical Sciences and Peking Union Medical College, Beijing, 100021 China; Department of Obstetrics and Gynecology, Peking Union Medical College Hospital, Chinese Academy of Medical Sciences and Peking Union Medical College, Beijing, 100730 China

**Keywords:** PD-L1, Immunohistochemistry, *CD274* gene alternation, TNBC, Prognosis

## Abstract

**Purpose:**

To estimate the therapeutic potential of PD-L1 inhibition in breast cancer, we evaluated the prevalence and significance of PD-L1 protein expression with a validated antibody and *CD274* gene alternation in a large cohort of triple negative breast cancer (TNBC) and correlated with clinicopathological data and patients overall survival.

**Methods:**

Immunohistochemistry and in situ mRNA hybridization was used to detect PD-L1 protein and mRNA expression in tumor tissues from 183 TNBC patients respectively. Fluorescence in situ hybridization analysis was performed on PD-L1 strong expression samples to assess copy number on chromosome 9p24.1 of *CD274* gene.

**Results:**

Expression of PD-L1 by immune cells was observed in 4.9 % of TNBC, while expression by tumor cells accounted for 8.7 %. There was a high concordance in PD-L1 protein expression and *PDL1* mRNA expression. Samples with PD-L1 strong expression were found to have a *CD274* gene copy number gain. PD-L1 expression was correlated with higher tumor grade, but was independent of menopausal status, lymph nodes metastasis, histological subtype and tumor size. In addition, we used precise stratification of PD-L1 expression on tumor or immune cells of certain breast cancer subtype and suggested that patients with PD-L1 expression in basal-like tumors by immune cells or with *CD274* gene copy number gain had a longer disease-specific overall survival.

**Conclusions:**

Our findings may promote the more precise analysis of PD-L1 expression in breast cancer and aid the selection of patients who may benefit from immune therapy.

## Background

Immune responses are fine-tune regulated through a combination of stimulatory and inhibitory molecules and signal pathways (Dunn et al. 2002). To generate efficient antitumor immune responses by cytotoxic T lymphocytes (CTLs), inhibition of negative immune checkpoint proteins such as cytotoxic T-lymphocyte-associated protein (CTLA4), programmed cell death 1 (PD-1) and programmed death ligand 1 (PD-L1) are applied in recent clinical studies and trails (Brahmer et al. 2012; Topalian et al. 2012; Herbst et al. 2014). PD-L1, which belongs to B7 family, binds PD-1 and CD80 as counter receptors to offer negative signals that control and suppress CTL responses in both autoimmune responses and evasion of tumor immunity (Dong et al. 1999; Butte et al. 2007). PD-L1 is often expressed by activated immune cells including T cells, B cells, myeloid dendritic cells (DCs), macrophages and myeloid-derived suppressor cells. In addition, PD-L1 has also been found to be expressed in tumor cells and tumor-infiltrating immune cells (Taube et al. 2012). Consequently, clinical trials of blocking monoclonal antibodies (mAbs) against PD-1 and PD-L1 in a variety of solid tumors show promising results and validate this pathway as a therapeutic target.

Triple-negative breast cancers (TNBC) are defined as tumors that lack estrogen receptor (ER), progesterone receptor (PR), or human epidermal growth factor receptor (HER2) expression. These tumors account for 10–20 % of all breast cancers and are often associated with lymphocytic infiltration, higher grade and are biologically more aggressive (Badve et al. 2011). Despite having higher rates of clinical response to chemotherapy, TNBC patients have a worse prognosis owing to limited treatment options and higher rate of distant recurrence (Haffty et al. 2006; Dent et al. 2007). TNBC can be further subdivided into basal-like breast cancer and non-basal-like breast cancer according to immunohistochemical marker panels (Gazinska et al. 2013). Previous studies have demonstrated that PD-L1 protein or *PDL1* mRNA is rarely expressed in breast tumors, relatively enriched in basal-like breast tumors (Ali et al. 2015; Sabatier et al. 2015; Soliman et al. 2014). However, there is still not a unanimous agreement on whether PD-L1 expression in immune cells or tumor cells is an independent negative prognostic factor in breast cancer.

Here, we analyzed PD-L1 protein expression with a validated antibody and in situ *PDL1* mRNA expression in 183 TNBC of Chinese female patients. We investigated the prevalence of PD-L1 expression in TNBC and correlated with clinicopathological data and patients survival.

## Methods

### Patient population

Clinicopathologic information of TNBC patients were retrospectively collected from the Department of Pathology, Cancer Hospital, Peking Union Medical College, Chinese Academy of Medical Sciences, Beijing, China. The study comprised women diagnosed with TNBC from January 1999 to December 2008. Basal-like breast cancer is defined as triple-negative tumors (lack of ER, PR and HER2 expression) with expression of cytokeratin 56 (CK56) and/or epidermal growth factor receptor (EGFR) as previously reported. The inclusion criteria were also determined as follows: primary operable breast cancer, no family history for breast or ovary cancer, no prior treatments before surgery, mastectomies, or lumpectomies specimens with sufficient tissue. Tissue microarrays (TMAs) were built as previously reported (Zhu et al. 2015). In brief, two tumor cores and one normal core of 1.0 mm diameter were taken from each case based on hematoxylin and eosin (H&E) staining. Then, TMAs were performed with immunohistochemistry (IHC), in situ mRNA hybridization and fluorescence in situ hybridization (FISH). The study was approved by the Institute Review Board of the Cancer Hospital, Chinese Academy of Medical Sciences. The methods were carried out in accordance with the approved guidelines. Each participant signed an Institutional Review Board approved informed consent in accordance with current guidelines.

### PD-L1 immunohistochemistry and scoring

A previous validated rabbit monoclonal antibody (clone SP142; Ventana, Tucson, AZ) was used in IHC on an automated staining platform (Benchmark; Ventana) using a concentration of 4.3 µg/ml. Tumor samples were represented by double 1.0-mm cores in TMAs. Stained slides were scanned using an Aperio Scanscope AT digital slide scanner. PD-L1 was scored as reported in previous studies (Haffty et al. 2006), where tumor and immune cells were attributed separate scores on a four-point scale as follows: 0 (no staining), 1+ (cytoplasmic and/or weak membranous staining in <10 % of the positive cells), 2+ (weak to moderate cytoplasmic and/or membranous staining in ≥10 % of the positive cells) or 3+ (strong cytoplasmic and/or membranous staining in ≥10 % of the positive cells). PD-L1 scores in patients with multiple specimens were based on the highest score.

### In situ mRNA hybridization

In situ detection of PD-L1 transcripts in TMA samples was performed using the RNAscope 2.0 High Definition-BROWN assay with in situ hybridization probes (Advanced Cell Diagnostics, Hayward, CA) as previous reported. Briefly, 5 μm sections were deparaffinized, boiled with preamplification reagent for 15 min, and submitted to protease digestion followed by hybridization for 7 h with target probes to human *PDL1* mRNA. Diaminobenzidine (DAB) staining was used to visualize signals in a bright field microscope. Stained slides were scanned using an Aperio Scanscope AT digital slide scanner.

### Fluorescence in situ hybridization

FISH analysis was performed on TMAs to assess copy number on chromosome 9p24.1 using the *ZytoLight*^®^ SPEC *CD274* (PD-L1)/*CEN 9* Dual Color Probe. Dual Color Probe is a mixture of a green fluorochrome direct labeled SPEC *CD274* probe specific for the *CD274* genes at 9p24.1 and an orange fluorochrome direct labeled *CEN 9* probe specific for the classical satellite III region of chromosome 9 (D9Z3) at 9q12. The *CD274* FISH results were analyzed according to the previous study (Ansell et al. 2015). Control probe ratio of at least 3:1 were classified as amplified, those with a probe ratio of more than 1:1 but less than 3:1 were classified as relative copy gain, and those with a probe ratio of 1:1 but with more than two copies of each probe were classified as polysomic for chromosome 9p. Slides were evaluated independently by two experts blind to the patient’s history and histological findings.

### Statistical analysis

Correlations between continuous and ordinal variables were assessed using Spearman’s rank correlation coefficient. Differences of patient characteristics and clinicopathologic factors in the two-dimensional cross-comparison were evaluated statistically by Pearson’s χ^2^ test or Fischer’s exact test. Statistical tests were two-sided, and *P* < 0.05 were considered significant. Specific overall survival (OS) was calculated from the date of diagnosis to the date of death from breast cancer. Follow-up was measured from the date of diagnosis to the date of last news for event-free patients. Survivals were calculated using the Kaplan–Meier method and curves were compared with the log-rank test. Statistics were carried out using SPSS software (version 16.0 of SPSS, Chicago, IL, USA).

## Results

### PD-L1 expression in TNBC

**Fig. 1:**
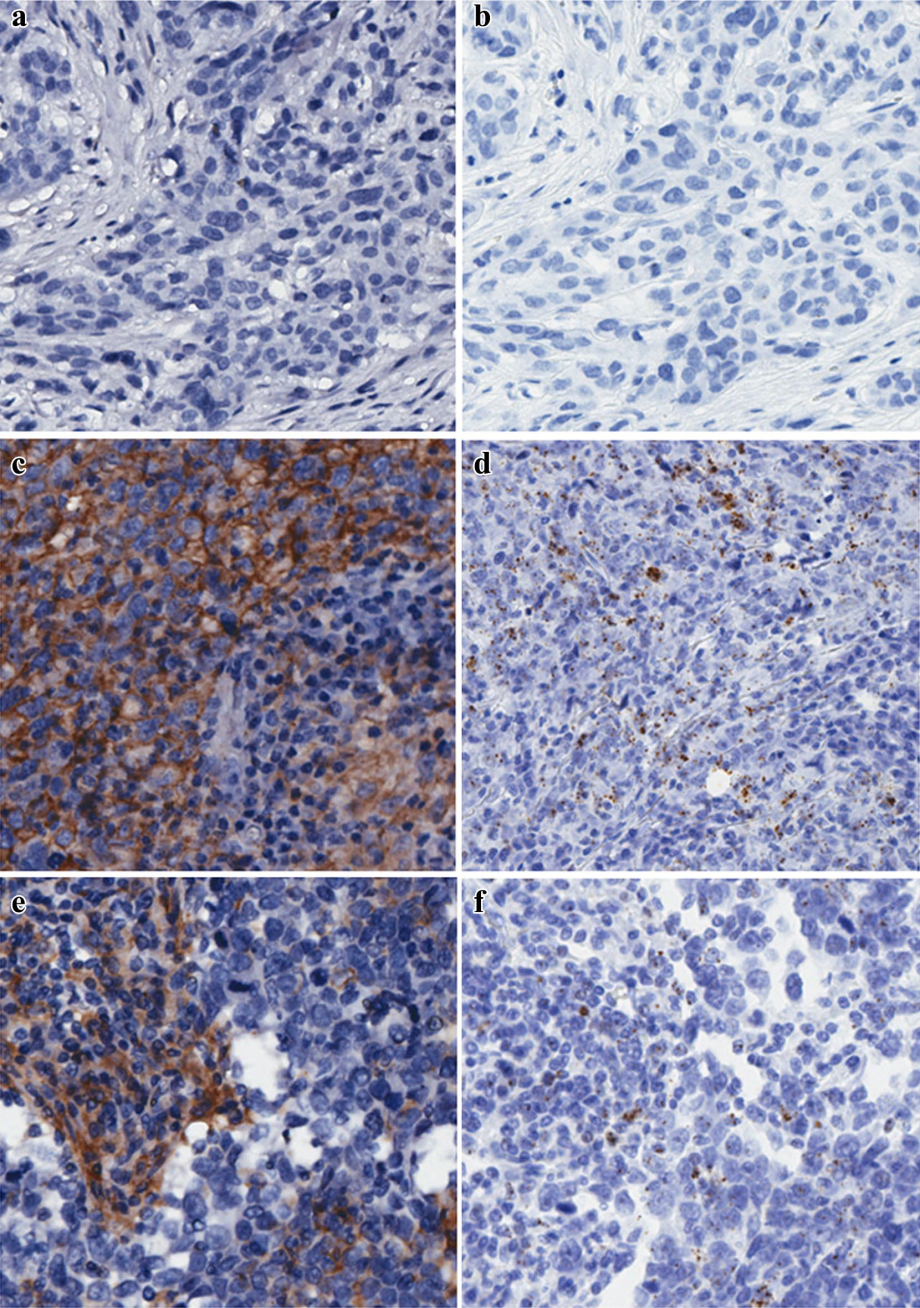
PD-L1 immunohistochemical and in situ mRNA expression in TNBC tissues. **a** PD-L1 negative expression with IHC detection (×200), **b** PD-L1 negative expression with in situ mRNA hybridization (×200). **c** Expression of PD-L1 protein in tumor cells (score as 3+) (×200). **d** In situ mRNA expression of PD-L1 is indicated by brown staining in tumor cells (×200). **e** Expression of PD-L1 protein in immune cells (score as 3+) (×200). **f** In situ mRNA hybridization of PD-L1 is indicated by brown staining in immune cells (×200)

**Fig. 2:**
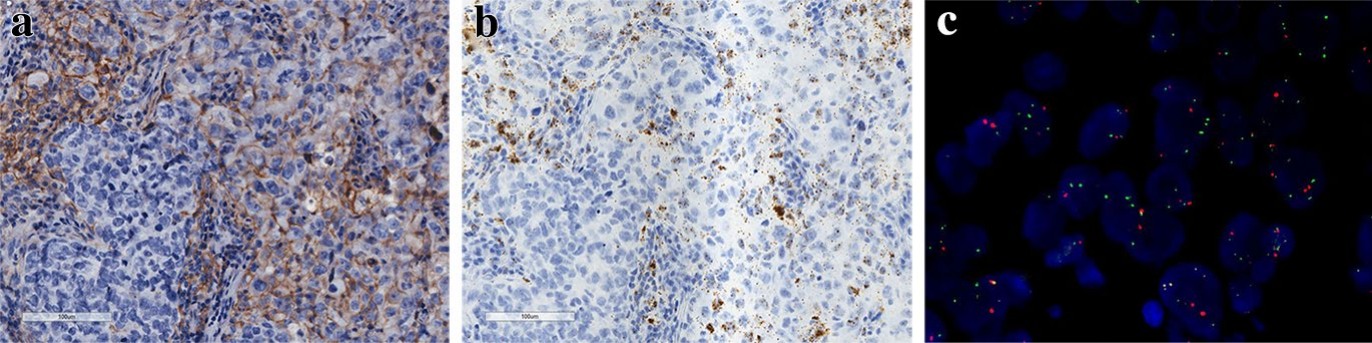
Immunohistochemical (IHC), in situ mRNA and fluorescence in situ hybridization (FISH) analyses of PD-L1 protein expression and *CD274* gene in patients with triple-negative breast cancer (TNBC). **a** Expression of PD-L1 protein in both immune and tumor cells (score as 3+) (×200); **b** In situ mRNA hybridization of PD-L1 from the same patient is indicated by brown staining (×200); **c** Representative image obtained from the same patient shows a copy number gain in *CD274* gene, with *CD274* (*green*) and *CEN9* (*red*) on chromosome 9p24.1 (×1000)

PD-L1 expression was measured by IHC and in situ mRNA hybridization (Fig. 1). There was a high concordance in PD-L1 protein expression and *PDL1* mRNA expression. In addition, seven samples with PD-L1 strong expression (score as 3+) were found to have a *CD274* gene copy number gain by detection of FISH (Fig. 2). Expression of PD-L1 in tumor and/or immune cells was observed in 13.7 % (25/183) TNBC tumors, where 16 tumors demonstrated basal-like breast phenotype. However, there was no significant difference in the proportion of PD-L1 expressed tumors between basal-like (n = 116) and non basal-like (n = 67) breast cancer subtype (13.8 vs 13.4 %, *P* = 0.94). Expression of PD-L1 by immune cells was observed in 4.9 % (9/183) of TNBC, while expression by tumor cells accounted for 8.7 % (16/183). Basal-like breast tumors showed 10.4 % (12/116) PD-L1 positive tumors cells, while 3.4 % (4/116) with PD-L1 positive immune cells.

### Clinicopathologic characteristics of PD-L1 positive TNBC

The clinicopathologic characteristics of TNBC stratified by PD-L1 expression was summarized in Table 1. Patients with PD-L1 expression demonstrated a slightly younger age than those with PD-L1 no expression, although this was not nominally statistically significant (64.0 vs 44.3 %, *P* = 0.09). Tumors with PD-L1 expression were more observed with higher tumor grade (G3) than PD-L1 no expression groups (96.0 vs 53.8 %, *P* < 0.0001). In addition, there was no significantly difference in aspects of menopausal status, lymph nodes metastasis, histological subtype and tumor size.

**Table 1:** Clinicopathologic characteristics and PD-L1 protein expression status

Characterics	PD-L1 expression (n = 25)	PD-L1 no expression (n = 158)	*P* value
Age (years, median)			0.06
<50	16 (64.0 %)	70 (44.3 %)	
≥50	9 (36.0 %)	88 (55.7 %)	
Menopausal status			0.57^‡^
Pre-menopausal	22 (88.0 %)	132 (83.5 %)	
Post-menopausal	3 (12.0 %)	26 (16.5 %)	
LNM			0.83
Present	8 (32.0 %)	54 (34.2 %)	
Absent	17 (68.0 %)	104 (65.8 %)	
Tumor grade			<0.0001^§^
G1	0 (0 %)	3 (1.9 %)	
G2	1 (4.0 %)	70 (44.3 %)	
G3	24 (96.0 %)	85 (53.8 %)	
Tumor type			0.25^§^
Ductal NOS	21 (84.0 %)	146 (92.4 %)	
ILC	0 (0 %)	1 (0.6 %)	
Other	4 (16.0 %)	11 (7.0 %)	
Basal-like subtype			0.94
Yes	16 (64.0 %)	100 (63.3 %)	
No	9 (36.0 %)	58 (36.7 %)	
Tumor embolus			0.32^‡^
Yes	1 (4.0 %)	21 (13.3 %)	
No	24 (96.0 %)	137 (86.7 %)	
Tumor size			0.06^†^
Mean (SD)	2.28 ± 0.91	2.74 ± 1.13	
Median	2.20	2.5	
Range	0.70–4.30	0.80–8.0	

*LNM* lymph nodes metastasis, *NOS* not otherwise specified, *ILC* invasive lobular carcinoma, *SD* standard deviation

^†^Two-sided Kruskal–Wallis test

^‡^Two-sided χ^2^ test with continuity correction

^§^Fischer’s exact test

Others are two-sided χ^2^ test

### Association with overall survival

**Fig. 3:**
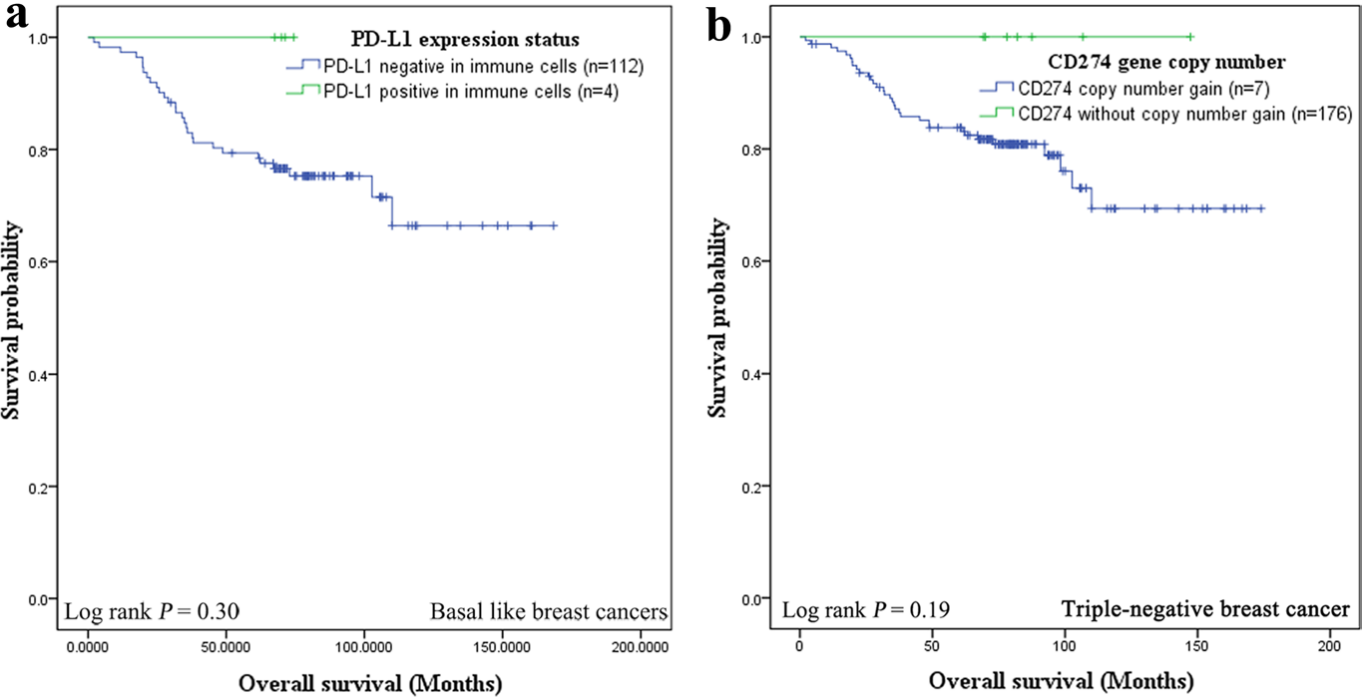
Kaplan–Meier survival curves of overall survival (OS) in patients with TNBC. **a** Kaplan–Meier graphical analysis of the OS in patients with basal-like tumors, a subgroup with PD-L1 expression by immune cells. **b** Kaplan–Meier graphical analysis of the OS in patients with *CD274* gene copy number gain

We assessed the prognostic value of PD-L1 expression in terms of OS. Overall survival data were available for 183 patients with a median follow up of 76.4 months (median OS not reached). The 5-year OS was 83.2 % (95 % CI 0.81–0.85). Expression of PD-L1, by either immune cells or tumor cells, was not significantly associated with outcome in neither TNBC, basal-like nor non basal-like groups. However, in basal-like tumors, a subgroup with PD-L1 expression by immune cells seemed likely to be associated with reduced disease-specific mortality although this was not statistically significant (*P* = 0.30, log-rank test, Fig. 3a). In addition, a trend of reduced mortality was also noted for TNBC patients with *CD274* gene copy number gain (*P* = 0.19, log-rank test, Fig. 3b).

## Discussion

We investigated the prevalence and pathologic characteristics of PD-L1 expression in a large cohort of TNBC breast cancer. Expression of PD-L1 by immune cells was observed in 4.9 % of TNBC, while expression by tumor cells accounted for 8.7 %. PD-L1 expression was correlated with higher tumor grade of TNBC, but was independent of menopausal status, lymph nodes metastasis, histological subtype and tumor size. Samples with PD-L1 strong expression were found to have a *CD274* gene copy number gain. In addition, there was a trend of longer disease-specific overall survival in basal-like tumors with PD-L1 expression by immune cells and TNBC patients with *CD274* gene copy number gain.

This is the first large cohort analysis of PD-L1 protein, in situ mRNA expression and *CD274* gene amplification in TNBC breast cancer. PD-L1 expression in breast cancer was reported in recent studies, however, the results varied regarding its expression rate and prognostic value (Ali et al. 2015; Sabatier et al. 2015; Qin et al. 2015; Schalper et al. 2014). In a study of 870 breast cancer patients, PD-L1 expression was observed in more than 20 % breast cancer and patients with TNBC seemed to have a higher proportion of positive PD-L1 expression rate of 55.9 % compared with other types (Qin et al. 2015). A previous study of 105 TNBC breast cancer revealed that 19 % (20/105) tumors exhibited PD-L1 expression (Mittendorf et al. 2014). In addition, Ali, et al. found that basal-like breast tumor, which is a subtype of TNBC, showed 19 % (56/302) PD-L1 expression in >1 % immune cells (Ali et al. 2015). These divergences could partially be attributed to the different antibodies used, notably in terms of specificity and reproducibility, and the IHC scoring system. Here, we used a validated PD-L1 rabbit monoclonal antibody of clone SP142 and a four-point IHC score scale which was proved to be effective in the previous studies (Herbst et al. 2014; Ali et al. 2015). We report that expression of PD-L1 by immune cells was observed in 4.9 % of TNBC, while expression by tumor cells accounted for 8.7 %. And basal-like breast tumors showed a total of 13.8 % PD-L1 expression either by immune cells or tumor cells. This was in accordance with two previous studies that less 20 % basal-like breast tumors exhibited PD-L1 expression (Ali et al. 2015; Mittendorf et al. 2014). In addition, previous observations also indicated that basal breast cancer cells constitutively express the high levels of PD-L1. Amplification of *CD274* has been observed in the setting of EBV-positive gastric cancer (Cancer Genome Atlas Research Network 2014) and in samples of Hodgkin’s lymphoma from patients who have had a clinical response to PD-1 inhibition (Ansell et al. 2015). Our results indicated that *CD274* gene was only slightly amplified in a small subset of breast cancer which showed strong protein expression of PD-L1.

Another focus of PD-L1 expression in breast cancer was notably on its prognostic value. A high expression of PD-L1 on tumor cells was associated with poor prognosis in several human maglignancies, such as non small-cell non cancer (NSCLC), melanoma and renal cancer (Azuma et al. 2014; Ott et al. 2013; Thompson et al. 2007). Especially, in a very recently published paper, Tao Qin’s work have revealed that patients with positive PD-L1 expression had significantly decreased survival compared to those with PD-L1 negative expression regardless of breast cancer subtype (Qin et al. 2015). However, other studies have opposite conclusions that PD-L1 protein or mRNA expression was associated with improved survival notably with basal-like breast tumors (Ali et al. 2015; Schalper et al. 2014). Furthermore, the improved survival was only observed in patients with a subgroup of basal-like tumors which PD-L1 expression was found in immune cells. These results indicated that the precise stratification of PD-L1 expression on tumor or immune cells of certain breast cancer subtype could promote the better understanding of its role on patients’ outcome. Our results suggested that in basal-like tumors, a subgroup with PD-L1 expression by immune cells seemed like to be associated with reduced disease-specific mortality. In addition, a trend of reduced mortality was also noted for TNBC patients with *CD274* gene copy number gain (*P* = 0.19) although this did not reach a significant difference due to limited sample size.

Patients diagnosed with TNBC have a higher risk of disease recurrence in all breast cancer subtypes (Le Du et al. 2015). Thus, identification and evaluation of new biomarkers and therapeutic agents is urgent for these patients. Because TNBC is a heterogeneous disease, the use of genome-wide association study may provide a rational for prognosis and prediction to therapy (Burstein et al. 2015; Lehmann et al. 2011; Prat et al. 2013). However, the biological classification based on gene expression profiles was until now unclear whether it could surely guide the targeted therapy. Anti PD-L1/PD-1 pathway therapy represents a promising cancer immune therapy method (Brahmer et al. 2012). Our results in addition with previous studies have strengthened the idea that PD-L1 is mainly expressed in TNBC of all breast cancer subtype. Through finely stratification of PD-L1 expression on TNBC, our observations suggested that PD-L1 inhibitors may also benefit a small subset of women with TNBC with tumors that express PD-L1.

The main limitation of this study was the use of TMAs for representation of tumors. In some tumors, immune infiltration may be heterogeneous and this heterogeneity will not be captured by TMAs. However, TMAs enable the conduct of large-scale pathology studies and in this way ultimately lead to more reliable conclusions.

To data, this is the first large study of PD-L1 expression in TNBC breast cancer. We found that PD-L1 expression was correlated with higher tumor grade of TNBC, but was independent of menopausal status, lymph nodes metastasis, histological subtype and tumor size. Samples with PD-L1 strong expression were associated with a *CD274* gene copy number gain. In addition, we used the precise stratification of PD-L1 expression on tumor or immune cells of certain breast cancer subtype and suggested that patients with PD-L1 expression in basal-like tumors by immune cells had a longer disease-specific overall survival. These findings may promote the more precise analysis of PD-L1 expression in breast cancer and aid the selection of patients who will surely benefit from immune therapy.
